# Ventilating Chambers

**Published:** 1877-04

**Authors:** 


**VENTILATING CHAMBERS.**—When it is considered that pure air is essential to the purification of the blood, and that the food we eat never becomes nutriment until it meets with the air in the lungs, and when it is furthermore remembered that a full third of our entire existence is passed in our sleeping apartments, it must be clear to the commonest understanding that the difference between breathing a pure and impure air while we are asleep is literally incalculable as to the effects upon our happiness and well-being.

